# Viruses, Autophagy Genes, and Crohn’s Disease

**DOI:** 10.3390/v3071281

**Published:** 2011-07-21

**Authors:** Vanessa M. Hubbard, Ken Cadwell

**Affiliations:** Skirball Institute of Biomolecular Medicine, Department of Microbiology, New York University School of Medicine, New York, NY 10016, USA

**Keywords:** Crohn’s disease, inflammatory bowel disease, norovirus, MNV, ATG16L1, autophagy, mucosal immunity, intestine, Paneth cells

## Abstract

The etiology of the intestinal disease Crohn’s disease involves genetic factors as well as ill-defined environmental agents. Several genetic variants linked to this disease are associated with autophagy, a process that is critical for proper responses to viral infections. While a role for viruses in this disease remains speculative, accumulating evidence indicate that this possibility requires serious consideration. In this review, we will examine the three-way relationship between viruses, autophagy genes, and Crohn’s disease and discuss how host-pathogen interactions can mediate complex inflammatory disorders.

## 1. Introduction

Crohn’s disease is a major form of inflammatory bowel disease that affects as many as 1 in 500 individuals [1]. Family and twin studies have demonstrated a strong heritable component to the disease that is the subject of intense research [2]. The age of onset is frequently in the second decade of life, and most patients progress to a relapsing disease characterized by abdominal pain, bloody diarrhea, vomiting, and weight loss. Crohn’s disease is distinguished from other types of inflammatory bowel disease such as ulcerative colitis in that the inflammation is often discontinuous, transmural, and can involve both the small intestine and colon [3,4]. Anti-inflammatory or immuno-suppressive drugs such as those that target TNFα can sometimes be effective, but surgical removal of inflamed regions is common despite medical intervention [5]. Additionally, significant complications such as colorectal cancer, malnutrition, and opportunistic infections in patients have been attributed to either chronic inflammation or aggressive medical treatment. A better understanding of disease origin and mechanism is necessary to improve the prognosis of this serious disorder.

Since the disease mechanism at some level involves an aberrant response to intestinal bacteria, there has been a significant attempt at identifying a bacterial pathogen that causes Crohn’s disease [6,7]. However, the difficulty in differentiating cause and effect of inflammation has prevented the identification of a single bacterial species that is clearly a trigger rather than an opportunistic infection [6,8]. Alternatively, it is possible that the genetic background favors the outgrowth of harmful bacteria that are already present in the gastrointestinal tract. Dramatic experiments in mice with *Klebsiella pneumoniae*, *Proteus mirabilis*, and adherent-invasive *Escherichia coli* (AIEC) have demonstrated that the increased presence of certain endogenous bacteria can induce intestinal disease [6,9,10]. This hypothesis is supported by the observation that the composition of intestinal bacterial species is altered in inflammatory bowel disease patients [11,12]. It is also possible that, instead of an outgrowth of certain pathogenic bacteria, harmless commensal species facilitate disease specifically in a genetically susceptible background. This concept is best demonstrated in dnKO mice (Tgfβ **d**ominant **n**egative x IL-10R2 **k**nock**o**ut) in which common *Bacteroides* species invoke fulminant colitis [13]. These studies collectively indicate that there are certain bacteria that can mediate disease, but that they are unlikely to be sufficient since these species are ubiquitous.

What are the genetic risk factors that could have such profound effects on the intestinal environment and host-bacteria interactions? Large-scale genetic studies of Crohn’s disease patients have successfully identified many susceptibility loci. The most recent meta-analysis of genome-wide screens confirmed the existence of 71 susceptibility loci [14], and many more are predicted to exist [15]. A striking example of the success of these studies is the association of several genes directly or indirectly implicated in the autophagy pathway. Autophagy is a major degradative pathway of the cell with several critical functions in innate and adaptive immunity [16]. As such, the relationship between autophagy and Crohn’s disease is an exciting area of research and progress. However, finer genetic mapping is necessary for some of these susceptibility loci that are near but not within the coding region of a gene. Moreover, statistical association of any gene with Crohn’s disease must be functionally validated, especially since most of the gene variants that have been identified confer a minimal degree of risk. As genome or exome sequences from patients become available, rare mutations that are inevitably missed by current approaches will be revealed [17]. Nevertheless, these genetic studies have provided critical evidence that Crohn’s disease is associated with numerous variants rather than a single mutation, and further characterization of susceptibility genes will surely increase our understanding of this disease and mucosal immunity in general.

**Figure 1:**
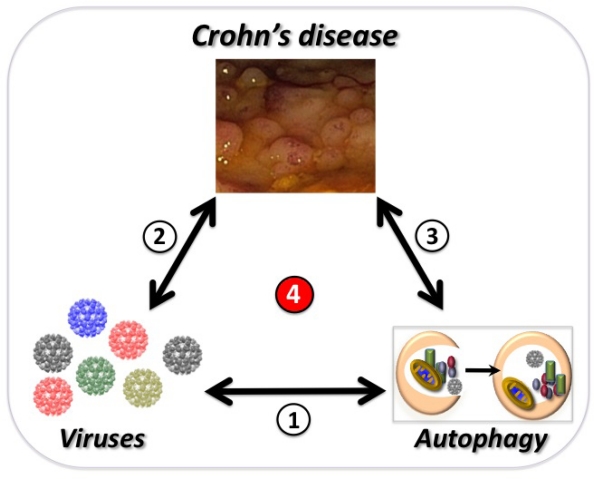
Overview of the three-way relationship discussed in this review. The relationship between autophagy and viruses (1) is the subject of this Special Issue of *Viruses*. The current evidence that viruses influence the course of Crohn’s disease (2) will be discussed in Section 2. Genes that participate in the autophagy pathway have been linked to Crohn’s disease (3) and will be discussed in Section 3. Finally, recent evidence in a mouse model linking all three (4) will be discussed in Section 4. The endoscopic image of the colon was taken from a Crohn’s disease patient and shows sigmoid pseudopolyps with cobblestone-like polyps.

While it is essential to identify gene variants and commensal bacteria that mediate intestinal disease, these approaches alone will be insufficient to explain the origin of Crohn’s disease. Many genes have unexpected cell-type specific functions that must be empirically derived. This concern is particularly germane for genes implicated in autophagy [16]. Additionally, there is a poorly understood environmental component to the disease that may precede or accelerate the inflammatory response to commensal bacteria. In light of recent evidence, this review will focus on the three-way relationship between viruses, autophagy genes, and Crohn’s disease (Figure 1). We will first examine the epidemiological evidence for a viral etiology of inflammatory bowel disease, a topic that has received inadequate attention due to the lack of available data. Then we will discuss the link between the autophagy pathway and Crohn’s disease including an unexpected interaction between a viral infection and the autophagy gene *ATG16L1*. Finally, we will revisit the role of commensal bacteria in the context of these recent findings and discuss why viruses and autophagy must continue to be examined specifically in Crohn’s disease.

## 2. Viruses and Inflammatory Bowel Disease

### 2.1. Evidence for an Infectious Trigger

Epidemiological studies have left no doubt that there is an environmental component to Crohn’s disease. Despite the identification of many genetic susceptibility loci, they collectively account for only a third of the disease risk [14]. Twin studies provide further confirmation that the genetic component is an important but incomplete explanation [2]. Additionally, as seen with other auto-inflammatory or autoimmune disorders, the incidence of inflammatory bowel disease has increased over the years in parallel with industrialization, with the highest incidences in Northern Europe and North America [1,18]. This geographical divide may be due to a differential exposure to certain classes of pathogens. While industrialization has encouraged the spread of certain food-borne viruses and bacteria [19], parasitic infections are still common in developing nations [20]. This correlative evidence is supported by both clinical studies and experiments in mice demonstrating that infection with a helminth such as *Trichuris* species protects against inflammatory bowel disease by suppressing inflammatory T cells [20–23]. Therefore, pathogens represent one group of environmental factors that are likely to be a key component to inflammatory bowel diseases such as Crohn’s disease.

### 2.2. Viruses Associated With Inflammatory Bowel Disease

Experiments reported in the 70s implicated a transmissible agent that passes through a 0.2 μm filter [24], but enthusiasm waned as subsequent studies failed to correlate specific viruses with the disease [25]. However, the techniques used to detect viruses have become significantly more sensitive, and several viruses have been linked by more recent association studies [26–30]. These viruses do not display exclusive association with Crohn’s disease and could be opportunistic or irrelevant as seen with cytomegalovirus (CMV). Infection with this β-herpesvirus is frequently asymptomatic and has an estimated prevalence between 30–100% worldwide [31]. CMV has received the attention of gastroenterologists because it can cause colitis in immuno-compromised individuals such as those infected with HIV or transplant recipients [32,33]. CMV reactivation from latency can be detected in inflammatory bowel disease patients, though reactivation is likely being triggered by inflammation rather than the other way around [34]. Similarly, the γ-herpesvirus Epstein-Barr virus (EBV) is highly prevalent and has been associated with inflammatory bowel disease because of its presence in the colonic tissue of patients [35]. A recent clinical study found that viral replication and proliferating B cells were increased in IBD patients [36]. Other herpesviruses have also been detected in patients, but like CMV and EBV, evidence of causality is lacking [30].

In a case report of an individual diagnosed with ulcerative colitis, Parvovirus B19 was detected in diseased tissue [27]. Parvovirus B19 is a non-enveloped single-stranded DNA virus that causes erythema infectiosum in children and is primarily spread by respiratory droplets [37]. Infection by this virus is common, but the suspicious presence in the inflamed intestinal mucosa described in this case report is novel. Another common virus that has been associated with inflammatory bowel disease are noroviruses, which are the leading cause of non-bacterial acute gastroenteritis [38]. The ease with which noroviruses spread in crowded areas and adapt to host immunity make them a natural suspect in intestinal diseases that are becoming more common like Crohn’s disease and irritable bowel syndrome [39–42]. Analyses of patient history suggest that acute gastroenteritis (not necessarily viral) precedes diagnosis of inflammatory bowel disease in some individuals [43–46], and there is evidence that noroviruses exacerbate inflammatory bowel disease [25,28]. Noroviruses are the only viruses that have been associated with Crohn’s disease in an animal model [47], a finding that will be discussed further. Higher power clinical studies will help determine if there is a genuine association between the above viruses and inflammatory bowel disease, but it is equally important to take advantage of advances in deep sequencing and bioinformatics to search for new viruses. The emphasis may initially be on viruses that infect the mammalian host, but these approaches are also likely to address the currently unknown role of the diverse set of bacteriophages present in the intestine [48].

### 2.3. Crohn’s Disease Genes and Viruses

Cigarette smoking, non-steroidal anti-inflammatory drugs (NSAIDs), and diet are all non-infectious environmental factors that can influence the course of inflammatory bowel disease [49–51]. Yet an infectious provocateur has remained the most satisfying environmental factor since pathogens are a strong evolutionary force that may have led to the selection of gene variants associated with inflammatory disorders [52,53]. The autophagy-related gene *IRGM* has undergone significant changes during evolution, and natural variants of this gene contribute to the response against *Mycobacterium tuberculosis* [54,55]. *IRGM* has been independently associated with Crohn’s disease [56]. Therefore, it is possible that certain aspects of the autophagy pathway are evolutionarily plastic and critical for a balanced immune response in the face of infectious threats that change over time.

Most of the genes associated with Crohn’s disease have known immune functions. *NOD2* was the first to be identified and encodes a cytosolic pattern recognition receptor (PRR) that induces NF-κB activation and cytokine expression in the presence of muramyl dipeptide (MDP), a derivative of bacterial peptidoglycan [57–59]. The mutations associated with Crohn’s disease are found in the Leucine rich repeat (LRR) domain that is essential for recognition of MDP [60]. Although it is unclear how these mutations contribute to intestinal disease and whether Nod2 binds MDP directly, these findings reinforce an important role for bacteria in Crohn’s disease. One group has found that single-stranded viral RNA can also bind Nod2, leading to its translocation to the mitochondria where it binds MAVS and signals type I interferon (IFN) production [61]. With the expanding role of Nod2, it will be important to validate these results and determine if Crohn’s disease mutations disrupt this pathway.

Many of the other genes linked to Crohn’s disease have been identified by genome wide association studies (GWAS) [14]. GWAS analyze variants, typically single nucleotide polymorphisms (SNPs), distributed throughout the genome and identify those that are represented more frequently in individuals with a trait or disease of interest. A number of these genes associated with Crohn’s disease are important for T cells. The SNPs in the *IL-23R* gene region display particularly strong association and significantly reduce the risk of developing Crohn’s disease [62]. IL-23 directs the differentiation of T_H_17 cells, pro-inflammatory T cells that produce IL-17, IL-22, and IL-21 [63]. Inflammatory bowel disease patients have high levels of IL-23, IL-17, IL-21, and IL-22 producing cells in their intestine [64–68] and T_H_17 cells are potent mediators of intestinal inflammation in mouse models of experimental colitis [69–71]. It is not difficult to imagine a scenario in which a viral infection would alter the total number and function of a subset of T cells that mediate chronic inflammatory disease. Although this concept has not been demonstrated specifically for T_H_17 cells and intestinal disease, observations made in *T-bet*/*Eomes*-deficient mice and patients infected with hepatitis C virus (HCV) and respiratory syncytial virus (RSV) indicate that it is a possibility [72–75].

The above description of *NOD2* and *IL23R* is far from complete, and there are many more putative susceptibility genes that have a more obvious role in antiviral immunity such as *IRF1* [14]. Again, many of these genes await further confirmation of their association with Crohn’s disease, and their significance in influencing disease course is uncertain. Before describing susceptibility genes related to the autophagy pathway, one more gene is worth mentioning in relation to viruses and Crohn’s disease. Unlike many of the gene variants examined in GWAS, the polymorphism in the fucosyl transferase gene *FUT2* linked to Crohn’s disease leads to a coding change with a clear effect [14,76]. Individuals that are homozygous for this ‘non-secretor’ variant do not secrete blood group antigens on epithelial surfaces and are significantly protected from symptomatic infection by certain norovirus strains [41,77]. Thus, this variant of *FUT2* links differential susceptibility to viral infection with risk of developing Crohn’s disease. These genetic associations do not necessarily implicate viruses but are compatible with a viral etiology or pathogen triggers in general.

## 3. Autophagy Genes and Susceptibility Loci: A Link Between Crohn’s Disease and Infectious Agents?

Autophagy is best known as a highly conserved cell survival mechanism that promotes general homeostasis by degrading damaged or unwanted proteins and organelles and providing nutrients during prolonged periods of starvation [78]. Since this pathway has been implicated in the maintenance of many immune cells including B and T cells [79–83], autophagy-deficiency could alter host immunity prior to the presence of a pathogen. Autophagy requires the generation of a *de novo* double-membraned vesicle (autophagosome) that sequesters and degrades encapsulated cargo via the eventual fusion with the lysosome. Intracellular pathogens are frequently observed within autophagosomes, and through a process often referred to as xenophagy, can be targeted for subsequent degradation much like an unwanted protein or organelle [16]. This simple mechanism does not always reflect what is occurring *in vivo*. Although cell culture experiments initially indicated that ICP34.5 encoded by herpes simplex virus (HSV-1) inhibits autophagy to evade intracellular degradation [84], later work established that this inhibition is important *in vivo* for evading antigen presentation and activating CD4+ T cells [85,86]. Also, the pathogenesis of Sindbis virus infection of the central nervous system indicates that autophagy can protect the host without altering viral replication, a mechanism that may be more relevant when discussing chronic inflammatory diseases [87].

The detailed discussion of how specific viruses negotiate the autophagy pathway is extensively discussed in the other reviews in this issue. Below, we have chosen to highlight several Crohn’s disease susceptibility genes because they have been demonstrated to have functions related to the autophagy pathway (Table 1). These genes are not necessarily essential for autophagy and can contribute to intestinal inflammation through unrelated mechanisms. Moreover, even genes that are essential for autophagy can participate in host defense in the absence of a double-membraned vesicle as seen with IFNγ-mediated destruction of *Toxoplasma gondii*, TLR-mediated phagosome maturation, and innate immune recognition of double-stranded DNA [88–90]. Therefore, the following genes are related to the autophagy pathway but can contribute to inflammatory bowel disease independently of an autophagosome.

### 3.1. ATG16L1

The first indication that the autophagy pathway contributes to Crohn’s disease came from three GWAS published close together that implicated a common Threonine to Alanine substitution (T300A) in *ATG16L1* [91–93]. *ATG16L1* encodes a protein that forms a complex with ATG5 and ATG12 that catalyzes and specifies the location of LC3 lipidation, an event that is essential for conventional autophagy [94–96]. *Atg16L1*^−/−^ mice die soon after birth similar to mice deficient in other essential autophagy genes [96]. Macrophages isolated from these mice display enhanced inflammasome activity and overproduce IL-1β and IL-18 in response to LPS [96]. As a result of this increased capacity to produce active IL-1β and IL-18, irradiated mice reconstituted with *Atg16L1*^−/−^ bone marrow show increased inflammation and lethality in response to dextran sodium sulfate (DSS) treatment, a model of chemically-induced colitis. Inflammasome activation can be enhanced by the increased reactive oxygen species (ROS) produced by damaged mitochondria that accumulate in the absence of functional autophagy [96–99], and a similar role for autophagy has been proposed in limiting type I IFN production downstream of viral infection [100,101].

Another *Atg16L1*-mutant mouse model was generated through gene-trap mutagenesis, a technique in which introduction of a false splice acceptor into an intron disrupts the expression of intact mRNA [102]. Mice with such mutations in the *Atg16L1* locus display decreased Atg16L1 protein levels and reduced autophagy but are viable [95]. These Atg16L1^HM^ (HM for **h**ypo**m**orph) mice have specific abnormalities in Paneth cells, specialized epithelial cells located at the base of the small intestinal crypts. Paneth cells are considered important for innate immunity as they package antimicrobial molecules into secretory granules, and they are also essential for maintaining the intestinal stem cell niche [103,104]. Paneth cells from Atg16L1^HM^ mice are morphologically aberrant, have defective granule formation and secretion, and overexpress inflammatory genes [95]. This observation in mice led to the finding that Crohn’s disease patients homozygous for the *ATG16L1* risk polymorphism display similar abnormalities in their Paneth cells, indicating that Atg16L1 has a conserved role in this unique cell-type [95].

Mice develop similar Paneth cell abnormalities when *Atg5* or *Atg7,* two other autophagy genes, are conditionally deleted in the intestinal epithelium [95,105]. These results indicate that this cell-type specific role of *Atg16L1* is related to the autophagy pathway and could be related to the damaged organelles that accumulate in mutant Paneth cells [95]. Although it is unclear if the mechanism involves conventional autophagy and autophagosomes, the effect of *Atg16L1*-deficiency on Paneth cells and the inflammasome could not have been predicted by population genetic studies. Other Crohn’s disease susceptibility genes are likely to have cell-type, organ, or context-specific functions that remain to be uncovered.

It is currently uncertain how the T300A substitution affects ATG16L1 function. This coding change occurs in a region that is not evolutionarily conserved and does not appear to be necessary for conventional autophagy [106,107]. In cell lines, *ATG16L1 T300A* impairs capture of *Salmonella* by autophagosomes, which suggests that this variant compromises xenophagy without altering starvation-induced autophagy [106,108]. Further investigation will be necessary since these results were not replicated by another study using primary mouse fibroblasts [107].

### 3.2. NOD2

Several groups have independently demonstrated that *Nod2* can function in the autophagy pathway in addition to signaling cascades, thus bridging two major Crohn’s disease susceptibility genes [108–111]. MDP activation of Nod2 induces Atg16L1-dependent autophagy, but not in cells expressing the disease variants of either of these susceptibility genes [108–110]. These studies demonstrate that this functional interaction between Atg16L1 and Nod2 is important for limiting intracellular bacterial replication as well as increasing MHC class II surface expression. Another group has shown that peripheral blood mononuclear cells (PBMCs) expressing the T300A risk allele of *ATG16L1* overproduce IL-1β in the presence of MDP, although expression of a NOD2 risk variant prevents this IL-1β production altogether [111]. In contrast to LPS-mediated overproduction of IL-1β in mouse *Atg16L1*^−/−^ macrophages, this process does not appear to involve enhanced inflammasome activity, and many outstanding questions remain.

These studies are immensely important because they begin to address the relationship between Crohn’s disease susceptibility genes. In the next step, such gene-gene interactions must be further examined in the setting of intestinal mucosal immunity. A commonly discussed model of Crohn’s disease is one in which individuals are genetically susceptible to a pathogen that triggers a compensatory and harmful immune response. Anti-bacterial autophagy through *Atg16L1*, *Nod2*, and potentially other autophagy-related genes is consistent with this model. However, one of the strongest experimental support for this model comes from an unrelated study using *Citrobacter rodentium* to induce intestinal inflammation in *Nod2*^−/−^ mice [112]. During *C. rodentium* infection, colonic stromal cells produce the chemokine CCL2 that is necessary to recruit inflammatory monocytes to the intestine. At first, *Nod2*^−/−^ mice experience less inflammation because *Nod2* is essential for the production of CCL2 by the stromal cells, and therefore inflammatory monocytes are not present. At later time points, the intestinal inflammation becomes significantly more severe and longer-lasting compared to control mice since the monocytes did not help clear the pathogen earlier [112]. At least superficially, this mechanism does not appear to be related to autophagy. Despite the well-performed cell culture experiments linking various Crohn’s disease genes to xenophagy or other autophagy-mediated processes, each of these genes must be thoroughly examined *in vivo*, especially in the context of infectious disease and commensal bacteria.

### 3.3. IRGM

*IRGM* is a member of the antimicrobial immunity-related GTPase family (also called p47 GTPases) family that can induce autophagy and was first linked to Crohn’s disease by GWAS [91,113]. Further mapping revealed a large 20 kb deletion of a regulatory region upstream of the coding region that is in strong linkage with the disease [114]. The effect of this deletion on the level of *IRGM* expression differs among various tissues and cell lines, thus underscoring the need for context-dependent experiments. Instead of an upstream deletion, a recent study suggests that a SNP in *IRGM* is the true Crohn’s disease risk factor [115]. The authors propose that this polymorphism prevents down-regulation by a microRNA (miR-196) that is highly expressed in Crohn’s disease intestinal tissue. They further demonstrate that greater IRGM expression leads to both co-localization of adherent invasive *E. coli* with the autophagy machinery and increased intracellular survival of the bacteria [115]. This observation opposes other studies in which IRGM increases autophagosome or phagosome maturation and decreases survival of intracellular bacteria [116–122]. Also, this and other strains of *E. coli* are more abundant in the mucosa of Crohn’s disease patients [123–129]. While inconsistencies within the literature must be addressed, the observation that a SNP could alter miRNA recognition is novel.

The ability of IRGM to induce autophagy and limit the replication of intracellular bacteria has been most extensively demonstrated with *Mycobacteria* [116–118,121]. Unexpectedly, IRGM enhances autophagy by inducing mitochondrial depolarization and can increase ROS production and cell death [118]. Its murine ortholog Irgm1 (Lrg-47) protects hematopoietic stem cells (HSCs) and CD4+ T cells from IFNγ-mediated cell death through a mechanism related to its autophagy function [121,122]. To relate these findings to Crohn’s disease, it will be critical to determine how human *IRGM* is regulated since it is not known to be IFNγ-responsive as seen with mouse *Irgm1* [130]. Nevertheless, *IRGM* could regulate inflammation by either regulating intracellular pathogens or cellular homeostasis much like *ATG16L1*.

### 3.4. XBP1

Unlike the other genes discussed in this section, the functional association of *X box-binding protein 1* (*Xbp1*) with intestinal inflammation came first and inspired analysis of its genetic linkage to inflammatory bowel disease. Xbp1 was identified as a transcription factor that regulates the expression of MHC-II and was subsequently shown to be an essential part of the unfolded protein response (UPR) pathway [131–134]. During ER stress, the Xbp1 messenger RNA undergoes Ire1-dependent splicing which is necessary for translocation into the nuclease where it regulates UPR gene expression [131–133]. Secretory cell-types appear to be particularly dependent on Xbp1 since the process of secretion places a high burden on the ER. *Xbp1* deletion prevents the proper function of immunoglobulin-secreting plasma B cells and digestive enzyme-secreting zymogenic cells in the stomach [135,136]. Also, *Xbp1*-deletion in the intestinal epithelium leads to apoptosis of secretory granule-producing Paneth cells and mucus-secreting goblet cells [137]. Remarkably, many of these mice display spontaneous small intestinal inflammation as well as a more severe response to DSS-induced colitis [137]. Based on these results in mice, the authors questioned whether human *XBP1* is mutated in inflammatory bowel disease patients. Deep sequencing of the *XBP1* locus in 1200 Crohn’s disease and ulcerative colitis patients identified several rare variants that confer hypomorphic expression *in vitro* [137]. This genetic linkage is consistent with other studies [138–140].

Considering the connection between ER stress and autophagy, it is interesting to note that Paneth cells are abnormal in the absence of both autophagy and *Xbp1*. ER stress triggers autophagy to maintain homeostasis, and inhibiting autophagy can lead to ER abnormalities [141–144]. Therefore, altered *Xbp1* and *Atg16L1* function can theoretically lead to intestinal disease through the same mechanism. However, *Xbp1* deletion leads to Paneth cell death, an outcome that is more severe and distinct when compared to *Atg16L1* hypomorphism or *Atg5/Atg7* deletion [95,105,137]. For these reasons, functions of Xbp1 that are unrelated to autophagy will also be of interest when investigating intestinal inflammation.

### 3.5. Toll-Like Receptors

Toll-like receptors (TLRs) are a class of transmembrane receptors that recognize a variety of conserved structures found in microbes. Several risk variants in *TLR1*, *2*, *4*, *5*, *6*, and *9* have been described, though these genes have not been associated with inflammatory bowel disease in the comprehensive genome wide association studies. One explanation for this discrepancy is that *TLR* variants influence severity and symptoms rather than predicting the general risk of developing disease [145]. For instance, ulcerative colitis patients are more likely to present inflammation that spreads throughout the large intestine if they carry certain polymorphisms in *TLR1* and *2* [146]. A detailed discussion is beyond the scope of this review, but there is no doubt that multiple TLRs have essential functions in homeostasis, cancer, and immunity in the gastrointestinal tract [147–150]. Local stimulation of TLRs in the intestine with viral or bacterial ligands can have a profound effect on systemic antiviral immunity [151]. TLRs also have a complex relationship with the autophagy pathway that could be important in intestinal disease. Like NOD2, stimulation of some TLRs such as LPS-activation of TLR4 can induce autophagy [152], and as previously mentioned, autophagy genes regulate the function of TLR4 and other TLRs [89,96,153]. Since the molecular mechanism is becoming apparent in some examples [154], it will be possible to disrupt the intersection between TLRs and autophagy to test specific models. Collectively, this large body of literature indicates that TLRs integrate signals from multiple sources in the intestinal environment and shapes immunity at the cellular level.

### 3.6. Other Autophagy-Related Genes

The following autophagy-related genes have been tentatively linked to inflammatory bowel disease by one SNP in a non-coding region. The first two are associated with autophagy due to their potential involvement in trafficking of the autophagosome. *Leucine rich repeat kinase 2 (LRRK2)* is associated with Crohn’s disease through a GWAS meta-analysis [155] but a nearby gene (*MUC19* which encodes a mucin) is as likely to be the gene of interest. Mutations in *LRRK2* are implicated in Parkinson’s disease (PD) [156,157], possibly due to a role for this gene in autophagy-mediated clearance of protein aggregates in neurons [158]. Deletion of *LRRK2* or expression of Parkinson’s disease mutants leads to increased autophagosomes and protein aggregates [158,159]. One study strengthened the argument that *LRRK2* is a Crohn’s disease gene by demonstrating that this gene is regulated by IFNγ and expressed in immune cells in the mucosa of patients [160]. A SNP near *VAMP3*, which encodes a V-SNARE, is also a risk factor for Crohn’s disease [14]. In the autophagy pathway, VAMP3 was reported to mediate the specific fusion of multi-vesicular bodies (MVBs) with autophagosomes [161]. Further functional examination of this protein could yield a general role in cellular immunity since VAMP3-positive compartments are involved in phagocytosis of HIV and *Mycobacteria* [162,163]. The autophagy pathway requires many of the same proteins utilized by other membrane trafficking events [164,165], and understanding how *LRRK2* and *VAMP3* regulate autophagy or mucosal immunity appears important regardless of their significance in Crohn’s disease.

The three remaining genes influence autophagy through upstream signaling events. Signal transducers and activators of transcription (*STAT3*) is associated with Crohn’s disease by a meta-analysis of GWAS [14] and encodes a nuclear transcription factor that regulates gene expression downstream of several cytokine and growth factor receptors that are critical for immunity including IL-23R [166]. A siRNA screen revealed that STAT3 limits autophagy in an mTOR independent fashion [167], and autophagy may be involved in STAT3 activation as well [168]. Death associated protein (*DAP* or *DAP1*) has been linked with ulcerative colitis rather than Crohn’s disease [169], and encodes a ubiquitously expressed protein originally identified in a screen for factors involved in IFNγ-induced apoptosis [170,171]. Recently, DAP1 was shown to be phosphorylated and inhibited by mTOR under nutrient rich conditions. In contrast, during nutrient starvation when mTOR is inactive, DAP1 becomes dephosphorylated and acts as a negative regulator to prevent uncontrolled autophagy [170,171]. Finally, a recent study specifically examining autophagy gene loci found a SNP in *Unc-51-like kinase-1 (ULK1)* to be present more frequently in Crohn’s disease patients [172]. ULK1 is suppressed by mTOR, but unlike DAP1, signals the activation of autophagy as part of a larger complex [173,174]. It will be important to determine if these are *bona fide* susceptibility genes since signaling events upstream of autophagy may be easier to target pharmacologically.

## 4. Virus-plus-susceptibility Gene Interaction in Crohn’s Disease

### 4.1. Common Pathogens Alter Disease Penetrance in Mice

A novel association between a virus, an autophagy gene, and Crohn’s disease was revealed when the aforementioned Atg16L1^HM^ mice were transferred to an enhanced barrier facility [47]. Before discussing the details, it is first necessary to understand the context in which this experiment was performed. Differences in rodent animal facilities are a notorious variable exemplified by studies on the role of IL-10 in intestinal disease. IL-10 is a pleiotropic anti-inflammatory cytokine expressed by several different cell-types [175–178]. Mice deficient in IL-10 or a subunit of the IL-10 receptor display highly variable spontaneous intestinal inflammation, and the severity depends on the animal facility in which they are kept [175]. One reason why inconsistencies exist between different animal facilities is due to the presence of one or more infectious agents. In support of this hypothesis, intestinal disease in IL-10-deficient mice can be exacerbated by intentional infection with *Helicobacter* species that are commonly found in mouse colonies [179].

In addition to *Helicobacter*, the widespread prevalence of murine norovirus (MNV) has become evident. MNV was discovered when investigators observed that *Stat1*^−/−^ *Rag2*^−/−^ double mutant mice succumbed to death when tissue homogenates were injected intra-cerebrally [180]. Sequencing analysis of the infectious material revealed the presence of a positive-sense RNA virus related to noroviruses that infect humans, viruses that are responsible for the majority of non-bacterial gastroenteritis [181]. It is likely that MNV was present in the mice at the onset of these experiments despite being kept in an animal facility where pathogens are actively monitored. After its discovery, multiple strains of MNV have been identified and shown to be present in many mouse colonies worldwide [182–185].

**Figure 2:**
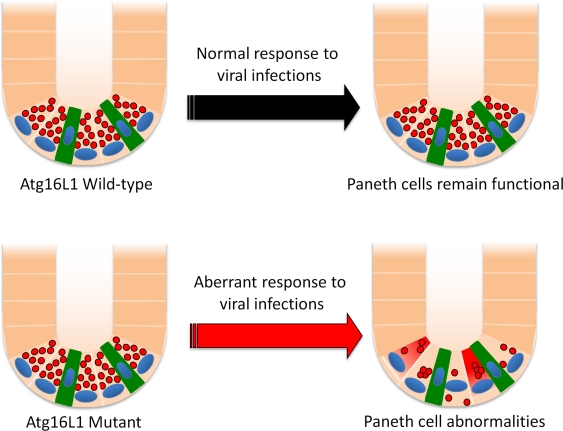
*Atg16L1* mutation leads to Paneth cell abnormalities during viral infection. The small intestinal crypt contains several Paneth cells with antimicrobial granules (red circles) and intestinal stem cells (green). The response to murine norovirus (MNV) infection leads to Paneth cell abnormalities specifically in *Atg16L1* mutant mice including depletion of granules, aberrant morphology, and altered gene expression. In contrast, MNV does not cause these changes in control mice.

MNV has become a critical model for investigating the biology of noroviruses since the human viruses do not establish a productive infection in a genetically-tractable small animal model. However, the presence of MNV in mouse colonies has become a concern for those examining mucosal immunity especially because many strains persistently infect the gastrointestinal tract [185]. Atg16L1^HM^ mice were originally housed in a barrier facility known to harbor MNV (as it happens, the same facility in which the virus was discovered). To differentiate between experimental results that are dependent and independent of the presence of MNV, the Atg16L1^HM^ mice were re-derived into an MNV-free enhanced SPF facility by embryo transfer. Surprisingly, re-derived Atg16L1^HM^ mice do not display any of the Paneth cell abnormalities described in mice raised in the previous facility [47]. Upon oral inoculation with the laboratory strain MNV CR6, Atg16L1^HM^ but not control mice develop Paneth cell granule abnormalities as well as an altered gene expression profile (Figure 2). Interestingly, many of the genes that display differentially altered expression after 7 days of infection are associated with intracellular protein trafficking or amino acid metabolism, two processes related to autophagy. Since MNV infects macrophages and dendritic cells rather than the intestinal epithelium [47,186,187], one potential mechanism is that autophagy-deficiency renders Paneth cells sensitive to soluble factors released during MNV infection. As previously mentioned, a sophisticated stress response may be necessary to support granule production by this specialized cell-type, and autophagy is best known as a pathway to maintain cellular homeostasis.

### 4.2. An Autophagy Gene and a Virus Interact to Generate Intestinal Disease

In addition to abnormalities in Paneth cells, MNV CR6 also mediates an aberrant intestinal injury response in Atg16L1^HM^ mice [47]. Dextran sodium sulfate (DSS) is a chemical that is commonly used to assess the intestinal injury response. In contrast to uninfected Atg16L1^HM^ mice or MNV-infected control mice, Atg16L1^HM^ mice infected with MNV CR6 develop several pathological hallmarks of Crohn’s disease after DSS treatment including villus blunting in the small intestine and transmural inflammation in the colon. Neutralizing TNFα or IFNγ, two cytokines central to Crohn’s disease [188,189], can prevent this abnormal intestinal injury specific to MNV-infected Atg16L1^HM^ mice. These pathologies are also prevented by broad-spectrum antibiotics treatment. These results indicate that the mechanism by which this virus-plus-susceptibility interaction generates disease in this model utilizes factors that are also important in human Crohn’s disease such as pro-inflammatory cytokines and commensal bacteria.

This interaction between MNV CR6 and *Atg16L1* mutation is remarkably specific. It is unlikely that all enteric infections will have this effect in Atg16L1^HM^ mice since another strain of MNV (MNV CW3) does not trigger any of the Crohn’s-like abnormalities attributed to MNV CR6 [47]. These two strains of MNV display 95% amino acid sequence identity across the genome. One explanation for this strain-specificity is that unlike MNV CR6, MNV CW3 is unable to establish a persistent infection. Therefore, it is possible that a continual immune response against an enteric virus is what mediates these Crohn’s-like pathologies. Why this aberrant immune response requires *Atg16L1* mutation is a critical question that must be addressed. Like the example with Sindbis virus [87], MNV replication is not altered in Atg16L1^HM^ mice, indicating that intracellular degradation of virions is not the explanation. Further investigation of the host response to MNV is likely to reveal novel mechanisms by which *Atg16L1* contributes to immunity.

### 4.3. Interpreting the Interaction Between MNV and Atg16L1

The MNV-*Atg16L1* interaction provides a specific example of how a pathogen and a susceptibility gene can combine to generate inflammatory bowel disease. Such results in a model organism do not necessarily indicate that an enteric virus directly triggers Crohn’s disease in humans. Without further evidence, we must resist the temptation to associate an agent that causes acute gastroenteritis with a disease that features chronic gastroenteritis. Epidemiological studies that have looked at an association between infectious gastroenteritis and inflammatory bowel disease show promising results but are far from conclusive [25,28,44–46]. Nonetheless, the ability of MNV to trigger Crohn’s-like pathologies in a mouse model reinforces the need to carefully examine a potential role of viruses in Crohn’s disease patients. A major role of viruses may explain why antibiotics and probiotics have had limited success in treating patients.

The most important lesson from the Atg16L1^HM^ mice is not the potential identity of a virus in Crohn’s disease, but the possibility that an infectious trigger is genotype-specific. MNV CR6 infection does not lead to Paneth cell and DSS-induced abnormalities in control mice and many other mutant mouse strains [47,190]. If these results reflect the pathophysiology of human Crohn’s disease, then an individual pathogen or gene may display only poor association with disease incidence and severity. Additionally, while the end result (intestinal inflammation) could be similar among patients, the route towards disease may differ between individuals. For instance, mice deficient in the Crohn’s disease gene *Nod2* develop intestinal pathologies after infection with *Helicobacter hepaticus* or *Citrobacter rodentium* [112,191]. Although the effect of MNV CR6 in these mice need to be examined, it is possible that different Crohn’s disease susceptibility genes interact with unrelated pathogens to mediate intestinal pathologies. In this disease model, finding one specific causative agent would be difficult. Since different Crohn’s disease patients have a distinct set of gene variants, it stands to reason that patients may have been exposed to a distinct set of pathogens as well.

## 5. Commensal Bacteria

### 5.1. Crosstalk Between MNV and Bacteria

Any model in which a virus triggers or exacerbates inflammatory bowel disease must consider the large body of work implicating bacteria in the disease [6]. In the above example in which MNV induces abnormalities in *Atg16L1* mutant mice, the virus mediates disease through commensal bacteria [47]. Viral infection could alter the composition of commensal bacteria, potentially by inhibiting the ability of Paneth cells to secrete antimicrobial molecules. The Atg16L1^HM^ model would be a useful system to determine if a specific bacterial species or intestinal bacteria in general mediate disease downstream of a gene-environment interaction. The answer to this question has practical implications for the use of antibiotics to treat inflammatory bowel disease. There is already evidence that genotype can alter responsiveness to antibiotics during recurrent Crohn’s disease [192].

Another relevant observation in a mouse model is that MNV infection promotes inflammation and lethality to secondary bacterial infection [193]. In this study, type I IFN produced in response to MNV infection potentiates Nod1 and Nod2 activity that leads to an exaggerated and harmful response to *E. coli*. Additionally, co-infection with MNV and *Helicobacter bilis* exacerbates intestinal inflammation in *Mdr1a*^−/−^ mice [194]. *Mdr1a* encodes a transmembrane transporter expressed by intestinal epithelial cells, and *Mdr1a*^−/−^ mice develop spontaneous intestinal inflammation possibly due to an inability to clear toxins [195]. The effect of MNV infection alone in *Mdr1a*^−/−^ mice has not been carefully examined, but this observation is consistent with the ability of MNV to mediate inflammation in conjunction with bacteria. Co-infection with MNV and *Helicobacter* species is likely a common occurrence in mouse colonies, and yet spontaneous intestinal inflammation is rare. The evidence thus far suggests that MNV has evolved to innocuously co-exist with the intestinal bacterial flora unless host immunity is compromised by secondary bacterial challenge or genetic deficiency, in which case virus-bacteria interactions can become pathogenic. This property of MNV resembles the selective ability of *Helicobacter* and *Bacteroides* species to mediate intestinal disease in specific genetic backgrounds [13,179]. The hypothetical viral, bacterial, parasitic, or fungal pathogens that mediate Crohn’s disease may have similarly co-evolved with humans.

### 5.2. Relationship Between Viruses and Bacteria in Intestinal Disease

Are there other examples in which commensal bacteria mediate disease downstream of a viral infection? Although experimental evidence of this concept may be new to Crohn’s disease research, crosstalk between viruses and bacteria in other pathogenic situations have been documented. Chronic immune activation is a characteristic abnormality associated with HIV infection, the degree of which is strongly correlated with disease progression [196]. Systemic immune activation stems from circulating microbial products that are derived from commensal bacteria that breach the intestinal epithelium [197]. A breakdown in intestinal barrier function is a hallmark of inflammatory bowel disease [198] and a realistic mechanism by which a virus could trigger intestinal disease; HIV infection is frequently associated with gastrointestinal dysfunction [199]. Additionally, changes in the composition of commensal bacteria can be detected in macaques that develop colitis after infection with simian immunodeficiency virus (SIV) [200]. These pioneering observations with HIV and SIV necessitate similar experiments with other viruses.

In addition to an effect of viral infection on commensal bacteria, the composition of commensal bacteria can influence the pathogenicity of a virus. Depletion of gram-positive bacteria with neomycin diminishes transcription of IL-1β/IL-18 and the subsequent adaptive immune response to influenza [151]. A remarkable aspect of this observation is that other antibiotics (vancomycin, ampicillin, or metronidazole) have a significantly less pronounced effect indicating that certain bacteria are more important than others for supporting antiviral immunity. Since influenza infection can lead to a catastrophic secondary infection with *Streptococcus pneumoniae* [201], it will be equally important to examine if influenza infection can alter the host’s ability to tolerate intestinal bacteria.

### 5.3. Autophagy and Commensal Bacteria

Observations made in the Atg16L1^HM^ mouse model provide an *in vivo* example of a crosstalk between the autophagy pathway and commensal bacteria. *In vitro* experiments also indicate that there is such a relationship. A modest increase in *adherent-invasive E. coli* replication can be detected during siRNA-mediated knock-down of *Atg16L1* [120], raising the possibility that autophagy deficiency could favor the expansion of certain bacteria in the intestine. The autophagy pathway could also be important in dampening inflammatory responses to non-invasive intestinal bacteria. In addition to LPS, increased production of IL-1β in *Atg16L1*^−/−^ macrophages can be induced with commensal bacteria such as *E. coli*, *Enterobacter aerogenes*, and *Klebsiella pneumonia* [96]. This abnormal cytokine response to these common gram-negative bacteria may explain why chimeric *Atg16L1*^−/−^ mice or MNV-infected Atg16L1^HM^ mice are susceptible to DSS treatment [47,96]. The increasing evidence linking the autophagy pathway to Crohn’s disease should motivate investigators to further examine the role of autophagy genes in controlling endogenous intestinal bacteria.

## 6. Concluding Remarks

We have summarized the current epidemiological, genetic, and experimental evidence supporting a role for viruses and/or autophagy in Crohn’s disease. However, it is important to avoid generalizations—a mechanism that applies to one Crohn’s disease patient may not be valid for another patient. We predict that if viruses that trigger Crohn’s disease exist, this will only be true for individuals of a certain genetic background as seen in the Atg16L1^HM^ example in mice. This hypothesis is consistent with the opposing roles of *Nod2* and *Atg16L1* downstream of viral infection [47,193]. Similarly, whether it is xenophagy, organelle homeostasis, or a non-canonical function, the contribution of the autophagy pathway to Crohn’s disease will only apply to a subset of individuals. The promise of individual-based therapy relies on understanding individual-based disease mechanism.

With this caveat in mind, we wish the reader to consider the following. Although Crohn’s disease patients may be susceptible to pathogens [202], this susceptibility is dissimilar in both extent and outcome when compared to truly genetically immuno-compromised individuals [203]. While autophagy can degrade intracellular pathogens in some situations, especially in cell culture, autophagy is definitely essential for maintaining cellular homeostasis in many physiological circumstances. We propose that if there is a relationship between autophagy and viruses in Crohn’s disease, then autophagy is most likely protecting the host against an over-exuberant response to a viral infection rather than directly suppressing virion production. If correct, then investigators should not expect to find a virus that consumes the host. Instead, an effort should be placed to search for signatures of an aberrant response to a viral infection, perhaps a response that can be targeted therapeutically.

## Figures and Tables

**Table 1:** Genes related to the autophagy pathway associated with inflammatory bowel disease. The association between inflammatory bowel disease and the genes below the divider requires further validation. The right column indicates if a gene has been demonstrated to function in an antiviral response.

**Autophagy Gene**	**Function**	**Antiviral Response**
***ATG16L1***	Essential for autophagosome formation, suppressing inflammasome activity, and maintaining Paneth cells.	Yes
***NOD2***	Induces cytokine expression and autophagy in the presence of muramyl dipeptide from the bacterial cell wall.	Yes
***IRGM***	Small anti-microbial GTPase that regulates autophagy.	Yes
***XBP1***	Leucine rich repeat protein associated with Parkinson’s disease and autophagosome maturation.	?

***LRRK2***	Leucine rich repeat protein associated with Parkinson’s disease and autophagosome maturation.	Yes
***VAMP3***	V-SNARE involved in fusion of MVBs to autophagosomes and other membrane trafficking events.	Yes
***STAT3***	Transcription factor downstream of cytokine and growth factor receptors that can inhibit autophagy.	Yes
***DAP***	A substrate of mTOR that negatively regulates autophagy.	?
***ULK1***	A kinase that is part of a major regulatory complex directly upstream of autophagy.	?
***TLRs***	Immune receptors that recognize conserved structures found in pathogens. Their activity can sometimes depend on autophagy.	Yes
